# Activated protein C in epilepsy pathophysiology

**DOI:** 10.3389/fnins.2023.1251017

**Published:** 2023-10-13

**Authors:** Linda Ines Zoungrana, Steven Didik, Hao Wang, Lily Slotabec, Ji Li

**Affiliations:** ^1^Department of Surgery, Morsani College of Medicine, University of South Florida, Tampa, FL, United States; ^2^Department of Physiology and Biophysics, University of Mississippi Medical Center, Jackson, MS, United States

**Keywords:** epilepsy, activated protein C, seizure, neuroprotection, neurologic disorders

## Abstract

Epilepsy is one of the most common neurologic disorders that is characterized by recurrent seizures, and depending on the type of seizure, it could lead to a severe outcome. Epilepsy’s mechanism of development is not fully understood yet, but some of the common features of the disease are blood-brain barrier disruption, microglia activation, and neuroinflammation. Those are also targets of activated protein C (APC). In fact, by downregulating thrombin, known as a pro-inflammatory, APC acts as an anti-inflammatory. APC is also an anti-apoptotic protein, instance by blocking p53-mediated apoptosis. APC’s neuroprotective effect could prevent blood-brain barrier dysfunction by acting on endothelial cells. Furthermore, through the downregulation of proapoptotic, and proinflammatory genes, APC’s neuroprotection could reduce the effect or prevent epilepsy pathogenesis. APC’s activity acts on blood-brain barrier disruption, inflammation, and apoptosis and causes neurogenesis, all hallmarks that could potentially treat or prevent epilepsy. Here we review both Activated Protein C and epilepsy mechanism, function, and the possible association between them.

## Introduction

Epilepsy is one of the world’s oldest recognized neurological disorders and the 4th most common neurologic disorder after migraine, cerebrovascular disease (stroke), and Alzheimer’s disease (Johns Hopkins Medicine, 2020). Epilepsy, characterized by recurrent seizures, affects around 50 million people worldwide (Centers for Disease Control and Prevention, 2023). These seizures usually lead to the involuntary movement of part of the body or the entire body and could eventually lead to losing consciousness, temporary confusion, a staring spell, stiff muscles, uncontrollable jerking movements of the arms and legs, and abnormal movements (Sander, 2014; Centers for Disease Control and Prevention, 2023). There are two main types of epileptic seizures: generalized seizures, which affect the whole brain, and focal, or partial seizures, which affect just one part of the brain; temporal lobe seizures, or focal seizures, are the most common type of epilepsy (Centers for Disease Control and Prevention, 2020). The worst form of epilepsy is status epileptogenesis (SE), which is characterized by a seizure that is longer than 5 min or multiple seizures within a short period (Smith et al., 2018). This form of epilepsy required immediate attention as it could be life-threatening. Activated protein C (APC) is a natural anticoagulant protein that plays an important role in regulating blood clotting (Hayashi and Suzuki, 2015). It serves as a potent anticoagulant in the blood, mainly by inactivating coagulation factors Va and VIIIa (Alberelli and De Candia, 2014). This mechanism helps maintain blood fluidity and prevent excess production of blood clots (Alberelli and De Candia, 2014). In addition to its anticoagulant properties, APC also regulates the coagulation cascade, which ensures clot formation only when necessary (Shahzad et al., 2019). One of APC’s biological functions is its fibrinolytic properties (Reda et al., 2019). Indeed, by acting on plasminogen activator inhibitor-1 (PAI-1), APC promotes the breakdown of existing blood clot formation, therefore preventing clot propagation (Reda et al., 2019). Furthermore, APC can modulate the immune response by reducing leukocyte adhesion to endothelial cells, suppressing the production involved in inflammation, and inhibiting the release of pro-inflammatory cytokines (Kant et al., 2020). APC also helps maintain the integrity of endothelial cells lining the blood vessels, as those cells regulate vascular tone, blood flow, and prevent clot formation (Ren et al., 2022). Lastly, APC preserves the integrity of various barriers in the body, including the blood-brain barrier, has cytoprotective properties, and activates several cell signaling pathways, including the activation of the protein C receptor (PAR-1) (Griffin et al., 2015). APC has been the focus of a few studies in which its cardioprotective, anti-inflammatory, and anti-apoptosis properties have been proven (Legrand and Tolwani, 2021). However, recently, APC’s neuroprotective effects have been of research interest. Although epilepsy is one of the oldest neurological disorders, its mechanism of development is not fully understood. This review paper aims to highlight a few mechanisms involved in the induction and perpetuation of epileptic seizures. Furthermore, we will examine the role and mechanism of activated protein C. Additionally, we will explore the association between activated protein C and epileptic seizures.

### Blood-brain barrier dysfunction

Brain function and neuronal environment are maintained within a specific homeostatic range, which is regulated by the blood-brain barrier (BBB) (Swissa et al., 2019). BBB is an element of the neurovascular unit composed of neurons, microglia, astrocytes, pericytes, and cerebral vessels in addition to BBB, and deregulation of these microunits is present in neurodegenerative diseases such as Alzheimer’s disease or inflammatory-related diseases such as stroke or epilepsy (Rhea and Banks, 2019; Swissa et al., 2019). The BBB is composed of brain microvascular endothelial cells, the first interface between the blood and the brain (Persidsky et al., 2006). Those endothelial cells within the vessel function as osmoregulation, leukocyte trafficking, transport of nutrients, and a barrier, and to properly accomplish those functions, they have unique properties that include adherens junctions, tight junctions, and junctional adhesion molecules (Persidsky et al., 2006). The presence of adherens and tight junctions, or junctional adhesion molecules, composes the brain microvascular endothelial cells (Persidsky et al., 2006). Those elements have an increased number of mitochondria, which are essential for the transport of nutrients to the brain (Oldendorf et al., 1977; Persidsky et al., 2006). Enzymes such as aromatic acid decarboxylase, γ-glutamyl transpeptidase (γ-GTP), or alkaline phosphatase are present in high concentrations in the cerebral microvessels and metabolize bloodborne solutes, nutrients, and drugs, providing an enzymatic barrier (Abbott, 2005; Löscher and Potschka, 2005; Persidsky et al., 2006). These enzymes, as well as the polarity present between the abluminal and luminal surfaces of the brain microvasculature, provide a highly tightly regulated barrier (Persidsky et al., 2006). The extracellular matrix on which the endothelium cell lies also makes up the BBB structure. It provides anchors to the brain microvascular endothelial cells via collagen type IV, laminin, integrin, and other matrix proteins (Persidsky et al., 2006; Kadry et al., 2020). Disruption of the BBB extracellular matrix is associated with disordered development (Persidsky et al., 2006; Kangwantas et al., 2016). Next to the blood endothelial cell, there are the astrocytes that envelope the BBB endothelium, creating a tight interaction between them that influences their structure (Persidsky et al., 2006). Astrocytes represent the most abundant cell of the central nervous system (CNS), and whenever endothelial cells and astrocytes interact, the endothelial cell tight junctions are amplified, decreasing the gap in the area and increasing the number of astrocytic cells as well (Persidsky et al., 2006; Kadry et al., 2020). Astrocytes help maintain BBB function and tightness. In addition to astrocytes, pericytes are also part of the neurovascular unit, which plays an important role in BBB microvasculature stability, angiogenesis, and integrity (Peppiatt et al., 2006; Hall et al., 2014; Kadry et al., 2020). Due to their similar contractual ability to smooth muscle cells, pericytes can also control blood flow by regulating the capillary diameter (Sagare et al., 2013). Pericytes are closely connected to the endothelial cell and tight junction, which is unable to send cellular projection and react in the case of brain trauma or hypoxia (Dore-Duffy et al., 2000; Gonul et al., 2002; Persidsky et al., 2006). Pericytes regulate a few aspects of the neurovasculature (Heymans et al., 2020). For instance, Eilken et al. (2017) showed that the expression of vascular endothelial growth factor receptor 1 (VEGFR1) by pericytes could affect VEGF signaling, and depletion of pericyte activity could lead to an angiogenic defect, limited endothelial sprouting, and the enlargement of vessels (Persidsky et al., 2006). In summary, each of the components of the BBB plays an important role in the physiological aspect and function of the brain. Disruption of one of the components of the BBB could lead to neuroinflammation, neurological disorders, and neuronal hyperexcitability, including epilepsy. In fact, BBB association with epilepsy is not a new phenomenon, and decades of research have shown that BBB leakage can cause epilepsy and lead to status epilepticus.

### BBB dysfunction in seizure

Years of research on BBB dysfunction have been reported in humans after brain injury, status epilepticus, as well as in temporal lobe epilepsy (TLE) animal models, induced by pilocarpine, kainic acid, or the electrical stimulation-induced seizure model (van Vliet et al., 2007; Wang et al., 2012; Yan et al., 2018; Mendes et al., 2019). This means that BBB dysfunction can occur because of epileptic seizures. In the animal model study, BBB leakages were observed in various regions of the brain, including the cortex, hippocampus, thalamus and amygdala. An intense seizure could lead to a change in the brain’s electrical potential or reduce its electrical signaling (Dreier, 2011; van Vliet et al., 2015). These changes lead to a tone alteration of the blood vessel, which can lead to hypoperfusion or hyperperfusion and cause tissue damage (Winkler et al., 2012; van Vliet et al., 2015). Furthermore, this will lead to cellular damage and a decrease in blood pressure and pH, causing hypoxia and further enhancing BBB dysfunction (Stanimirovic and Friedman, 2012; van Vliet et al., 2015). SE has a high mortality rate, with survivors often experiencing complications that include epilepsy (Swissa et al., 2019). SE animal research has reported excitotoxicity, neuronal dysfunction, cell loss, and the development of epilepsy (Swissa et al., 2019). During the SE *in vivo* experimental model, a rapid increase in BBB permeability has been observed within the first 30 min in animals (Shrot et al., 2014; Swissa et al., 2019). In those research models, at 48 h, a quantifiable BBB leakage revealed that localized BBB dysfunction is highly sensitive to developing epilepsy on average 4 weeks later (Bar-Klein et al., 2017; Swissa et al., 2019). Whenever epilepsy was established, the histological analysis confirmed it with the presence of albumin, serum proteins, and IgG, as well as reactive microglia and astrocytes, neuroinflammation, and cellular damage (Swissa et al., 2019). These indicated SE animal models show BBB dysfunction and robust inflammatory response drugs that induce SE like, pilocarpine (Fujikawa, 1996; Tang et al., 2011; Swissa et al., 2019). The mechanism that underlies BBB dysfunction is not yet fully understood; however, there seems to be a close association between neuronal hyperactivity and BBB dysfunction (Milikovsky et al., 2017; Swissa et al., 2019). The extracellular level of glutamate is associated with SE, and glutamate, by binding with the brain endothelial cell, can alter tight junctions or reduce transcellular trafficking (Krizbai et al., 1998; Sharp et al., 2003; András et al., 2007; Swissa et al., 2019). The release and activation of glutamate also induce oxidative stress and increase intracellular calcium, which has been associated with increased BBB permeability (Brown and Davis, 2002; De Bock et al., 2013). Pericytes that have been found to secrete pro-inflammatory cytokines and actively participate in neuroinflammatory responses seem to be associated with BBB dysfunction during SE (Fabry et al., 1993; Armulik et al., 2010). Rearrangement and proliferation of pericytes were observed during epileptogenesis, and SE providing evidence of the association of pericytes with epilepsy (Klement et al., 2018; Swissa et al., 2019). After brain injury, BBB dysfunction is usually characterized by the extravasation of albumin circulating in the vessel (Swissa et al., 2019). Once in the extracellular space, albumin binds to the astrocytes through transforming growth factor beta receptors (TGF-βRs), causing Smad2/3 phosphorylation (Cacheaux et al., 2009). This phosphorylation leads to a transcriptional modification that results: (1) in a downregulation of the inward-rectifying potassium channel, which is responsible for maintaining the membrane resting potential and regulating the electrical excitation of neurons cells; (2) also a strong neuroinflammatory response with IL-1β, IL-6, and other pro-inflammatory cytokines being upregulated; (3) rearrangement and rewiring of the neuronal network, as well as synapse plasticity; (4) changes in the perineuronal microenvironment and upregulation of matrix metalloproteases (MMPs); and (5) excitatory synaptogenesis (Frigerio et al., 2012; Baronas and Kurata, 2014; Levy et al., 2015; Weissberg et al., 2015; Salar et al., 2016; Kim et al., 2017). These events are probably what enhance the seizure mechanism.

### Microglia and glia activation in seizure

Neuroglia, or glia cells, are the majority composed of astrocytes, oligodendrocyte lineage cells, microglia, as well as progenitors NG2-glia (Jäkel and Dimou, 2017). Over the years, researchers have presented and shown the importance of glia cells in the nervous system; however, there is still much to know. Astrocytes have multiple functions, but as previously mentioned, they play a role in BBB integrity (Persidsky et al., 2006). Oligodendrocyte lineage cells have multiple functions and help in the formation of myelin sheaths found on nerve axons, as well as supporting axons’ metabolic activity and neuroplasticity (Zhou et al., 2021). Progenitors NG2-glia are found in the white and gray matter of developing as well as the mature central nervous system. Little is known about their function, but they also have the ability to generate myelinating and non-myelinating cells, just like oligodendrocytes (Nishiyama et al., 2014). Microglia, the focus of this review, is known to be the immune cell of the CNS, capable of creating an inflammatory response; they play an important role in phagocytosis of debris and apoptotic cells; they provide neuronal support during development; they assist in synaptic organization; as well as neuronal excitability (Bachiller et al., 2018). Even though we have a lot of knowledge about microglial function under physiological conditions, little is known about the microglia’s structure and function under SE conditions. Pioneers in the field have found an association between microglia and SE through their activation in regions of the brain affected by SE induced by drugs such as kainic acid or pilocarpine (Vezzani et al., 2015; Hiragi et al., 2018). Once activated, microglial cells release proinflammatory cytokines, creating an upregulation of glutamate, hyperexcitability, and the neurodegenerative hallmarks of epilepsy (Hiragi et al., 2018; Zhao H. et al., 2018; Andoh et al., 2020). Microglia most likely contribute to epileptogenesis and progress to Campbell et al. (1993) and Probert et al. (1997) were among the first to associate brain inflammation with epileptogenesis from their studies on transgenic mice, which showed overexpression of the cytokines IL-6 and TNF-α (Campbell et al., 1993; Probert et al., 1997). Since researchers have focused on proinflammatory cytokines and provided a timeline for their release. For instance, De Simoni et al. (2000) published in early 2000 that cytokines such as TNF-α, IL-1β, and IL-6 expression were elevated in the hippocampus on the first day of electric stimulation SE induction in rat models (Hiragi et al., 2018). On the other hand, TNF-α, IL-1β, and IL-6 expression increased 3 days after pilocarpine-induced SE (Benson et al., 2015; Hiragi et al., 2018). It is important to mention that anti-inflammatory cytokines such as IL-10 and IL-4 were also increased in microglia in an epileptic brain (Hiragi et al., 2018). Toll-like receptors (TLR) also play a role in an epileptic brain; in fact, *in vitro* studies reported that microglia responded to TLR3, and TLR4 agonists lead to the production of cytokines (Olson and Miller, 2004; Hiragi et al., 2018). Gross et al. (2017) showed that a deficit in TLR3 reduces recurring seizures in pilocarpine-induced SE, and earlier, Maroso et al. (2010) recorded a reduction of acute seizures in KA-induced SE by blocking TLR4 activity (Maroso et al., 2010; Gross et al., 2017; Hiragi et al., 2018). Furthermore, the activation of TLR9 by microglia in the hippocampus can attenuate convulsive seizures, and a deficit in TLR9 aggravates seizure severity and leads to cognitive decline (Matsuda et al., 2015). Although a large majority of researchers agreed that microglial activation contributes to epileptogenesis through proinflammatory releases, other research showed microglia association with epileptogenesis without a proinflammatory signal. Zhao X. et al. (2018) by studying *Tsc1*Cx3cr1CKO mice, have shown microglia association with epileptogenesis without a proinflammatory signal (Hiragi et al., 2018; Kinoshita and Koyama, 2021). Elevated Mammalian target of rapamycin (mTOR) signaling has been observed in epileptogenic human and animal models, and tuberous sclerosis complex 1 (TSC1) is known to be a negative regulator of the mTOR pathway, so Zhao X. et al. (2018) used that to investigate the mTOR association with microglia and epilepsy (Chu-Shore et al., 2010; Franz and Capal, 2017). In their study, *Tsc1*Cx3cr1CKO mice had elevated mTOR signaling only in microglia cells, which exhibited an unusual increase in the activity of those cells, such as phagocytic activity. However, even though the expression of proinflammatory cytokines was elevated in the hippocampus of those mice, their microglia had a decreased expression of proinflammatory cytokines (Hiragi et al., 2018; Zhao X. et al., 2018). Interestingly, at 5 weeks, *Tsc1*Cx3cr1CKO mice develop spontaneous seizures, suggesting the upregulation of mTOR in microglia could induce a seizure and eventually lead to SE (Hiragi et al., 2018; Zhao X. et al., 2018). Some could argue that other glial cells could have played a role, like astrocytes. For instance, Bianco et al. (2005) showed that an increased ATP release observed during seizures from astrocytes induced IL-1β release from N9 microglial cells derived from mice’s brains (Bianco et al., 2005; Stansley et al., 2012; Hiragi et al., 2018). Fractalkine chemokines like CX3CL1 also play a role in the epileptic brain. Primarily expressed by neurons, it binds to a CX3CR1 receptor present on the surface of microglia, and in epileptic patients, its protein levels are increased (Hiragi et al., 2018; Wyatt-Johnson and Brewster, 2020). In the pilocarpine-induced SE rats’ model, CX3CL1 immunoreactivity increased within the first 3 h in the hippocampus region and decreases 3 days later, while CX3CR1 remained after the 3 days (Yeo et al., 2011; Hiragi et al., 2018). Although neuronal damage was reported 3 days after SE rescue, it was possible to give antibodies against CX3CL1 or CX3CR1 (Yeo et al., 2011; Hiragi et al., 2018). However, other studies suggested that CX3CL1 could decrease gamma-aminobutyric acid (GABA)-evoked currents in excitatory neurons, regulating the excitatory/inhibitory (E/I) balance of neural circuits in seizures (Xu et al., 2012; Roseti et al., 2013). However, no studies have reported a link during SE, and a lot remains to be studied. CXCR4 and CXCL12 seem to induce the microglial release of TNF-α as well as the astrocytic release of glutamate, leading to neuronal hyperexcitability, suggesting their possible contributions to epilepsy (Devinsky et al., 2013; Hiragi et al., 2018).

### Pro-inflammatory gene activation in seizure

In addition to microglia activation, other inflammatory molecules have been associated with SE. VEGF, as previously mentioned, plays an important role in angiogenesis and BBB permeability, but an elevated level of VEGF protein and VEGFR expression has been observed in an epileptic seizure (Mukhtar, 2020). Nicoletti et al. (2008) found that after SE stimulation, VEGF might have a neuroprotective effect against SE. VEGFR was found to be stimulated on neuronal cells and upregulation of VEGF was observed on glial cells a day after pilocarpine-induced SE, preventing neuronal cell death (Rigau et al., 2007). The molecular mechanism of VEGF neuroprotection during SE is not fully understood; however, researchers suggest that the induction of the intracellular phosphatidylinositol 3-kinase/Akt pathway might block caspase-3 function, preventing apoptosis and increasing cell viability (Sun and Guo, 2005). Thus, depletion of VEGF during SE might enhance neuronal deterioration. We previously mentioned that TLR4 activity plays an important role in SE. Some studies suggest that forkhead transcription factor 3 (Foxp3) attenuates TLR4 signaling and inflammation, which then inactivates NR2B-containing *N*-methyl-D-aspartic acid (NMDA) receptors (Wang et al., 2017). This suggests that Foxp3 plays an important role in epileptogenesis (Wang et al., 2017; Mukhtar, 2020). Plus, the hyperacetylated form of high mobility group box 1 (HMGB1) regulates pro-inflammatory cytokines like IL-Iβ, and like Foxp3, it interacts with TLRs, TLR2, and TLR4 with receptors for advanced glycation endproducts (RAGE), its role in SE is still not fully understood (Mukhtar, 2020). Balosso et al. (2014) suggest HMGB1 augmented NMDA activity enhances excitotoxicity and aggravates Kainic acid seizure induced through activation of TLR4 in neurons located in the hippocampus (Mukhtar, 2020). When discussing neuroinflammation and seizures, NF-κB signaling pathway is one of the most important players. In fact, by interacting with other molecules such as COX-2, mTOR, and mitogen-activated protein kinase (MAPK), it can interact with other molecules such as HMGB1, TNF-α, and IL-1 and activate TLR-4, TNF receptor (TNFR), and IL-1R which are major players in the neuroinflammation process, but how is it associated with SE (Wang and Chen, 2018)? Lubin et al. (2007) have reported that the inhibition of the NF-κB signaling pathway significantly decreased brain-derived neurotrophic factor (bdnf) protein expression and inhibitor kappa B alpha (IκBα), in which an increase level is observed during seizure activity, suggesting NF-κB pathway involvement in the upregulation of these transcripts during SE. With its direct or indirect interaction with other molecules NF-κB plays an important role in neuroinflammation as well as being associated with SE.

Mammalian target of rapamycin plays an important role in cellular mechanism, and as we also mentioned earlier, it is no surprise that its activity could be associated with SE. Genetic deficits of cellular elements in the mTOR pathway, like TSC, phosphatase, and tensin homolog (PTEN), are related to the development of epilepsy (Manning et al., 2002; Meikle et al., 2008; Zhou et al., 2009). It would explain that abnormal mTOR could result in SE. Previous studies have also reported that inhibition of the mTOR pathway could reduce seizures in SE and even restore BBB dysfunction, making it a potential treatment target for SE (van Vliet et al., 2016; Wang and Chen, 2018). Moreover, seizures could potentially activate NF-κB and other inflammatory molecules that could lead to SE (Wang and Chen, 2018; Mukhtar, 2020). MAPKs are composed of enzymes that play critical roles in the cellular response to various external stimuli and could be associated with SE (Wang and Chen, 2018). For instance, Yang et al. (2018) suggested that inhibition of p38 MAPK, a member of the MAPK family, could reduce the time to the first epileptic seizure and attenuate its severity in the pilocarpine-induced rat model of epilepsy. COX-2 and Prostaglandin E2 (PGE2) could lead to an increase in Ca^2+^, causing neuronal damage, a neurologic deficit, and hyperexcitability, possibly associating COX-2 and PGE2 with SE further studies are required (Wang and Chen, 2018; Mukhtar, 2020). Matrix metalloproteinase-9 (MMP-9) is a protease released by microglia in the hippocampus, cerebellum, and cortex part of the brain, it releases accelerated cell loss through disruption of matrix-cell, excitotoxicity, apoptosis, and BBB dysfunction, and its upregulation could lead to epileptogenesis (Acar et al., 2015; Bronisz and Kurkowska-Jastrzębska, 2016; Mukhtar, 2020). Furthermore, platelet-activating factor (PAF), CD44, and NADPH oxidases (NOXs) expression are increased during SE induction, affecting neuronal plasticity, hippocampal synaptic reorganization, or microglial activations, all of which enhance SE (Meikle et al., 2008; Zhou et al., 2009; Mukhtar, 2020).

### Protein C activation mechanism

**FIGURE 1:**
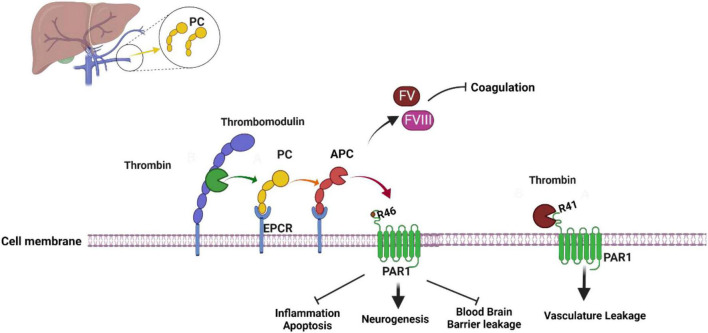
Activated protein C mechanism of activation and function. Thrombin-Thrombomodulin complex and endothelial protein C receptor (EPCR) are required to activate protein C to activated protein C which act as an anticoagulant. When activated protein C bind to protease activated receptor 1 (PAR1) it activated it neuroprotective mechanism. Thrombin interaction with PAR1 lead to vasculature leakage (see text for more information). The figure was prepared by software provided by Biorender.com (accessed 3 May, 2023).

Protein C (PC), a vitamin K-dependent serine protease zymogen, is a single-chain protein composed of a prepropeptide and a signal peptide and has its gene expression located on chromosome 2 (Plutzky et al., 1986; Brown et al., 2013). Synthesized in the male reproductive tract but mainly in the liver, it is a Ca^2+^- binding zymogen with its three domains: an N-terminal epidermal growth factor (EGF)-like domain, a γ*-*Carboxyglutamic acid-rich (GLA) domain, and a catalytic domain (Brown et al., 2013). PC circulates as a single-chain zymogen in plasma with a concentration of 4 μg/mL and is then activated by the thrombin-thrombomodulin complex (Griffin et al., 1982; Brown et al., 2013; Ren et al., 2019). In the form of PC, it does not have any physiological function; in order to function, PC needs to be converted to activated protein C (APC) (Brown et al., 2013). Thrombin is the physiological enzyme that converts PC in its zymogen form to APC in its activated form (Brown et al., 2013). When thrombin is bound to thrombomodulin (TM), it is more effective in activating PC to APC, but the complex thrombin-thrombomodulin is not the only one responsible for PC activation (Brown et al., 2013). Indeed, PC needs to bind to endothelial protein C receptor (EPCR) through its Gla-domain binds in order to be converted to APC; in fact, PC is activated by the proteolysis at Arg169 in endothelial protein C receptor (EPCR)-bound protein C by thrombomodulin-bound thrombin (Figure 1; Esmon, 1993; Fukudome and Esmon, 1994; Stearns-Kurosawa et al., 1996; Brown et al., 2013; Ren et al., 2019). In the form of APC, this protein has cytoprotective effect, anticoagulant, anti-inflammatory, and neuroprotective effect, making it a possible target for epilepsy treatment.

### Anticoagulant mechanism of APC

The most important defense mechanisms against bleeding include blood coagulation and platelet-dependent hemostasis (Dahlbäck and Villoutreix, 2005). The formation of platelets creates a plug that blocks the vascular lesion. At the same time, platelets are formed, and the coagulation mechanism is activated by tissue factor (TF) (Dahlbäck and Villoutreix, 2005). Once coagulation factor VIIa (FVIIa) binds to TF, it creates an FVIIa–TF complex that converts factor X (FX) and factor IX (FIX) to their active forms FIXa and Fxa (Schenone et al., 2004; Dahlbäck and Villoutreix, 2005). During the coagulation process, a very large amount of thrombin is generated as complex (FXa–FVa) converts prothrombin to thrombin, and thrombin can activate platelets, FVIII, FV, and convert fibrinogen to a fibrin clot, making thrombin an important procoagulant protein (Di Cera, 2003; Mann et al., 2003; Dahlbäck and Villoutreix, 2005). Blood coagulation is tightly controlled by anticoagulation proteins, and APC is one of them. One of APC’s targets in the regulation of the coagulation pathway is the inhibition of thrombin production through the inactivation of procoagulant cofactors FVa and FVIIIa (Figure 1; Dahlbäck and Villoutreix, 2005; Brown et al., 2013). With protein S and intact factor V, APC can regulate coagulation pathways. To inactivate cofactors FVa, APC cleaves the peptide bonds; Arg306-Asn307, Arg506-Gly507 and Arg679-Lys680 and to inactivate FVIIIa, APC cleaves Arg336-Met337, Arg562-Gly563, and Arg740–Ser741 (Dahlbäck and Villoutreix, 2005; Brown et al., 2013). Through these cleavages, APC switches factors FVa and FVIIIa from procoagulant to anticoagulant roles.

### Anti-inflammatory and cytoprotective effect

In addition to its anticoagulant and profibrinolytic functions, APC also has cytoprotective and anti- inflammatory properties. To activate APC’s protective activity, APC requires the Gla domain- dependent interaction with EPCR. This interaction gives APC the possibility to cleave the exodomain of protease-activated receptor 1 (PAR-1) leading to the activation of anti-inflammatory and cytoprotective signaling in vascular endothelial cells. PAR1, with the other members PAR2, PAR3, and PAR4 are G protein-coupled receptors (GPCR) from the large Rhodopsin family (Joyce et al., 2001; Pompili et al., 2021). To be activated, PAR1’s N-terminus, which contains a hirudin-like domain with a high-affinity binding site for thrombin, need to be cleaved (Vu et al., 1991; Pompili et al., 2021). In addition to thrombin, several proteases can cleave and activate PAR1, including APC, FXa, FVIIa, MMP2, MMP3, MMP8, MMP9, plasmin, trypsin, cathepsin-G, granzyme-A and B (Pompili et al., 2021). Thrombin cleaves PAR1’s N-terminus at Arg 41 leading to a conformational change of PAR1 and causing its coupling with multiple Gα proteins such as Gαi, Gαq, and Gα12/13 (Pompili et al., 2021). After being activated, PAR1 can recruit β-arrestin and activate Akt and Rac1 (Adams et al., 2011; Pompili et al., 2021). PAR1-Thrombin interaction initiated a pro-inflammatory mechanism that activated RhoA signaling pathway and phosphorylation of ERK1/2 (Mosnier et al., 2007, 2012; Ren et al., 2019). APC can also activate PAR1 by cleaving its N-terminus at Arg46 (Figure 1; Pompili et al., 2021). PAR1-APC interaction led to anti-inflammatory and cytoprotective mechanism activation. In fact, this activation leads to the downregulation of proapoptotic, and proinflammatory proteins such as NFκB or p53 and Bax, as well as the upregulation of antiapoptotic protein like Bcl-2 and anti-inflammatory proteins (Mosnier et al., 2007). The anti-inflammatory effect of APC involves its effect on leukocytes by limiting leukocyte adhesion and infiltration of tissues, maintaining vasculature integration, and inhibiting the release of pro-inflammatory cytokines and chemokines by leukocytes (Mosnier et al., 2007). Although APC activity is well known, its anti-inflammatory and cytoprotective mechanisms are not fully understood, creating limitations in the field.

### Neuroprotective effect of APC

Our group, Ren et al. (2022) and other researchers established the APC cardioprotective effect and showed APC can prevent cardiac damage during I/R-induced stress, but recently the neuroprotective effect of APC has been a focus in the research field. In addition to its anticoagulant and cardioprotective effects, APC has a neuroprotective effect against neuropathology such as multiple sclerosis, ischemic stroke, and traumatic brain injury and the activation of the neuroprotective mechanism involves APC-PAR1 as well as PAR3 interaction (Griffin et al., 2018). Indeed, just like APC can induce a non-canonical activation of PAR1 by cleavage at Arg46 (Figure 1), APC can induce a non-canonical activation of PAR3 by cleavage at Arg41. Through their activation, they lead to signaling activation that would lead to stabilization of the BBB, up-regulation of neuronal antiapoptotic and anti-inflammatory proteins, as well as neurogenesis (Griffin et al., 2018). However, the detailed mechanism of the APC neuroprotective effect remains unclear, requiring more studies. Although APC’s primary use has been to treat sepsis, its potential beneficial effects in the context of stroke, have been the focus of some research. Indeed, APC anti-inflammatory properties could be beneficial in reducing inflammation that occurs after stroke (Huuskonen et al., 2022). In fact, inflammation exacerbates brain injury after a stroke, and APC can help mitigate it (Lazic et al., 2019; Huuskonen et al., 2022). As mentioned above, APC has anticoagulation properties, and in an ischemic stroke caused by a blood clot, APC helps prevent it and improve blood flow in the affected area (Hall et al., 2014; Griffin et al., 2015; Huuskonen et al., 2022). APC antiapoptotic properties could limit cell death in stroke affected areas, regulate apoptosis, and inhibit cell death processes (Huuskonen et al., 2022). BBB is also one of the APC targets. In fact, APC helps maintain the integrity of the BBB, which could help reduce secondary damage following a stroke (Majid et al., 2020; Huuskonen et al., 2022; Wang et al., 2022). Lastly, APC can improve cerebral perfusion, and its neuroprotective effect can help reduce secondary damage following a stroke and reduce brain damage (Huuskonen et al., 2022; Wang et al., 2022). Despite those potential benefits, more research is needed to fully understand the effect of APC on stroke and to determine if it could be an effective treatment. As of right now, stroke treatment focuses on established therapies like thrombolytic drugs [like tissue plasminogen activator (tPA)], and due to its properties, it is understandable why APC could be a potential treatment target (Dong et al., 2019; Lazic et al., 2019).

### Epilepsy and APC

**FIGURE 2:**
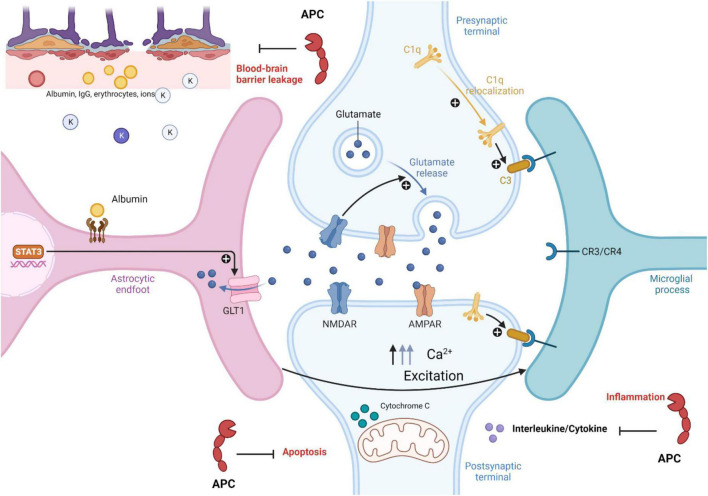
Role of activated protein C in epilepsy pathophysiology. Activated protein C neuroprotection in epilepsy pathophysiology inhibited blood brain barrier leakage, apoptosis, and inflammatory mechanism in disease development (see text for more information). The figure was prepared by software provided by Biorender.com (accessed 3 May, 2023).

Currently, in the field, there is still a lot to uncover regarding APC neuroprotective effects and the mechanism of epilepsy development; however, with the current knowledge, it is possible to make a connection between APC neuroprotective effects and epilepsy development. As mentioned earlier in this review, one of the hallmarks of epilepsy is BBB dysfunction, leading to reactive microglia and astrocytes, neuroinflammation, and cellular damage (Swissa et al., 2019). It is possible that the APC neuroprotective effect could prevent or treat epilepsy by initiating signaling effects on cells that could stabilize endothelial barrier functions like BBB, so in the presence of APC (Figure 2), BBB integrity remains intact (Griffin et al., 2018). As mentioned, inflammatory mediators could increase neuronal excitability and stimulate astrocytes and microglia activation during epilepsy, and since APC limited the release of pro-inflammatory cytokines and chemokines (Figure 2) by leukocytes in the vasculature, it could have an effect on microglia activation, limiting neuroinflammation during seizures (Mosnier et al., 2007; Kant et al., 2020; Mukhtar, 2020). In humans, neuronal cell death is one of the hallmarks of temporal lobe epilepsy. For instance, Bischoff et al. (2012) reported the death of interneurons in the pilocarpine seizure-inducing model and how Galectin-1 (Gal-1), a downstream effector of p75NTR, which triggers the disintegration of axons and cell death, plays a role in this mechanism (Bischoff et al., 2012). APC neurogenesis ability could reduce the effect of neuronal cell death through the replacement or creation of new neurons, particularly in the hippocampal region (Griffin et al., 2015, 2018). APC is not the only key element in its neuroprotective effect; PAR1 also plays an important role in this mechanism. PAR1 is in different regions of the brain, so its interaction with APC and other proteases could both be beneficial to the development of epilepsy or prevent it (Griffin et al., 2015, 2018; Heuberger and Schuepbach, 2019). For instance, Junge et al. (2003) reported in experimental brain ischemia that PAR1 activation by thrombin potentiates NMDA receptor responses and causes apoptosis in neurons, contributing to the pathological process (Junge et al., 2003). As outlined above, NMDA activity enhances excitotoxicity in epilepsy (Wang et al., 2017; Mukhtar, 2020). This suggests that increased interaction between PAR1 and thrombin might enhance seizure; however, APC-PAR1 might activate the neuroprotective mechanism (Griffin et al., 2018). There is still a lot to uncover about APC and the epilepsy association, even though APC has been proven to be an effective treatment target. In fact, in clinical studies, Wt-APC and 3K3A-APC have been given as treatments for ischemic stroke, sepsis, acute lung injury, diabetic ulcer wound healing, and more, and maybe with more research and a better understanding of their function, they could be used as a treatments or preventive methods for epilepsy.

## Summary

Epilepsy is one of the world’s oldest recognized neurological disorders and is characterized by recurrent seizures. A few of the mechanisms of development involved BBB disruption, neuroinflammation, and microglia activation. APC, well known for its anticoagulant properties, also has neuroprotective effects that could protect the BBB, activate anti-inflammatory and anti-apoptosis mechanisms, and lead to neurogenesis, all of which could help prevent or protect against epilepsy. In this review, we provided an overview of the possible association between APC and epilepsy, and a better understanding of both mechanisms is necessary to develop future therapies for current neurodevelopmental disorders.

## Author contributions

LZ and JL conceptualized and wrote the review and carried out literature analysis. HW, SD, and LS reviewed and edited the article. JL acquired funding. All authors contributed to the article and approved the submitted version.
